# The impact of the FilmArray meningitis/encephalitis panel on empiric antibiotic prescriptions in patients with suspected community-acquired meningitis

**DOI:** 10.1017/ash.2024.343

**Published:** 2024-07-26

**Authors:** Aaron Pathak, Caitlynn Pham, Sabra Shay, Todd Lasco, Mayar Al Mohajer

**Affiliations:** 1 School of Medicine, Baylor College of Medicine, Houston, TX, USA; 2 Department of Medicine, Baylor College of Medicine, Houston, TX, USA; 3 Department of Clinical Intelligence, Premier Inc., Charlotte, NC, USA; 4 Department of Pathology & Immunology, Baylor College of Medicine, Houston, TX, USA; 5 Department of Medicine, Section of Infectious Diseases, Baylor College of Medicine, Houston, TX, USA

## Abstract

The BioFire® FilmArray® meningitis/encephalitis (FA/ME) panel provides rapid testing for common cerebrospinal fluid pathogens. We compared empiric antibiotic utilization between patients with suspected community-acquired meningitis with and without an FA/ME panel ordered. No significant differences in antibiotic use were found.

## Introduction

Broad-spectrum antibiotics are recommended in patients with suspected community-acquired bacterial meningitis while waiting for cerebrospinal fluid (CSF) culture results to rule out life-threatening disease.^1^ However, excess use of empiric therapy has led to selective pressure on bacteria and the development of third-generation cephalosporin resistance in *Streptococcus pneumoniae*.^2,3^

Enhancing antibiotic stewardship is essential in preventing the increase of drug-resistant bacteria.^4^ The BioFire® FilmArray® meningitis/encephalitis (FA/ME) panel (BioFire Diagnostics, LLC, Salt Lake City, UT) identifies pathogens in community-acquired meningitis in < 1 hour, allowing clinicians to deescalate empiric antibiotics sooner than traditionally waiting for CSF culture results.^5^

Previous literature has revealed mixed results on antimicrobial usage despite the shortened detection time.^6,7^ A systematic review of ten studies showed an equal split of studies on the panel’s effect on antimicrobial usage.^7^ Furthermore, a meta-analysis of 13 studies found no significant difference in antibiotic days of therapy (DOT).^6^ Most of the included studies also focused on pediatric populations; only 3/10 of the studies included in the systematic review and 5/13 of the studies included in the meta-analysis focused on the adult population, limiting generalizability to adults.^6,7^

Given the limited literature among adult patients, this study aimed to evaluate the impact of the FA/ME panel compared to CSF culture alone on empiric antibiotic utilization in patients with suspected community-acquired meningitis.

## Methods

Our retrospective study included patients seen at three hospitals in Southeast Texas (one academic and two community centers) who received empiric antibiotics between 2017 and 2023 for suspected community-acquired meningitis. Patients were included if they underwent a lumbar puncture within 96 hours of admission and had a CSF Gram stain and culture obtained. Patients with ventricular drains, traumatic brain injury, and non-central nervous system infections were excluded. Cases comprised patients with an FA/ME panel performed, although controls included patients without the panel. The panel was available for clinicians in all three centers without restriction or education on the panel.

Primary outcomes included length of therapy (LOT) and DOT for empiric antibiotics. LOT was defined as the number of days a patient received empiric therapy with vancomycin or linezolid with one of the following: a third or fourth-generation cephalosporin, aztreonam, or trimethoprim-sulfamethoxazole. DOT was defined as the summation of days of each antibiotic prescribed for empiric treatment for suspected bacterial meningitis.

The Mann-Whitney and Fisher’s exact tests were used to compare differences in baseline characteristics between cases and controls. Two multiple linear regression models were applied to assess the relationship between the FA/ME panel use and study outcomes. Independent variables included were demographics, institution type, hospital unit, clinical signs and symptoms, CSF values, and FA/ME panel use. Imputation for missing values was performed using multiple imputation by chained equations. R version 4.2.2 (R Foundation for Statistical Computing, Vienna, Austria) was used for statistical analysis. This study was approved at our institution under Institutional Review Board protocol H-51640.

## Results

193 patients were included in our study (169 in the academic center and 24 in the non-academic centers). 71 patients (cases) received the FA/ME panel (along with CSF culture), although 122 patients received the CSF culture alone (controls).

Patients who received the FA/ME Panel were more likely to be in the academic center, admitted to the intensive care unit (ICU), had a seizure, had higher CSF protein, or had a negative Gram stain (Table 1). The median empiric LOT in cases and controls was 1.71 days and 1.18 days, respectively (Mann-Whitney, *P* = .160). The median DOT of cases and controls were eight and six days, respectively (Mann-Whitney, *P* = .045). Two patients had positive FA/ME panels, HSV1 and HSV2, respectively. Eight patients had positive CSF cultures, six in the cultures alone group and two in the FA/ME Panel group, neither of which had positive FA/ME Panels. These two positive CSF cultures were for Aspergillosis and coagulase-negative *Staphylococcus*, pathogens not included in the panel.

**Table 1.** Baseline characteristics for included patients with suspected bacterial meningitis

	FA/ME panel not performed (*n* = 122)	FA/ME panel performed (*n* = 71)	*P*
Age (years) – median (IQR)	53.03 (32.6–64.8)	54.3 (38.9–65.1)	0.515
Gender: Female – *n* (%)	75 (61.5)	41 (57.8)	0.649
Race: White – *n* (%)	75 (61.5)	37 (52.1)	0.129
Institution: academic – *n* (%)	101 (82.8)	68 (95.8)	**0.011**
Location			
Emergency department – *n* (%)	40 (32.8)	17 (23.9)	**0.018**
Intensive care unit – *n* (%)	35 (28.7)	35 (49.3)	
Floor – *n* (%)	47 (38.5)	19 (26.8)	
Presence of any comorbidity – *n* (%)	15 (12.3)	3 (4.2)	0.075
Fever – *n* (%)	55 (45.1)	38 (53.5)	0.297
Headache – *n* (%)	57 (46.7)	30 (42.3)	0.653
Altered mental status – *n* (%)	61 (50.0)	31 (43.7)	0.456
Neck stiffness – *n* (%)	15 (12.3)	6 (8.5)	0.479
Seizure – *n* (%)	13 (10.7)	19 (26.8)	**0.005**
Focal neurologic deficits – *n* (%)	18 (14.8)	17 (23.9)	0.124
CSF WBC – median (IQR)	2 (1–34)	2 (1–35)	0.923
CSF glucose – median (IQR)	65.5 (54.0–80.8)	66.0 (54.0–92.5)	0.377
CSF protein – median (IQR)	40.0 (25.0–73.0)	51.5 (37.0–97.0)	**0.007**
Positive CSF gram stain – *n* (%)	7 (5.7)	0 (0)	**0.048**
Positive CSF culture – *n* (%)	6 (4.9)	2 (2.8)	0.713
Positive FA/ME panel	N/A	2 (2.8)	N/A

Note. FA/ME, meningitis/encephalitis; IQR, interquartile range; CSF, cerebrospinal fluid; WBC, white blood cells.

Adjusting for confounders, the FA/ME panel did not impact the LOT (B = .13, *P* = .754, Table 2) or DOT (B = 1.15, *P* = .198). Patients with CSF collected on a non-ICU floor had higher LOT (B = 1.52, *P* = .002, Table 2) and DOT (B = 4.06, *P* < .001) compared to patients in the emergency department. Patients with a white blood cell count greater than five were associated with a longer LOT (B = 1.34, *P* = .001, Table 2) and DOT (B = 3.31, *P* < .001). A positive CSF gram stain was associated with longer DOT (B = 2.94, *P* = .01, Table 2) and LOT (B = 6.76, *P* = .007).

**Table 2.** Multivariate predictors of duration of empiric therapy and days of therapy

	Length of therapy (days)			Days of therapy (days)		
Predictors	Estimates	CI	P	Estimates	CI	P
(Intercept)	2.23	0.32–4.15	**0.022**	5.71	1.52–9.91	**0.008**
FA/ME panel performed	0.13	–0.68–0.93	0.754	1.15	–0.61–2.92	0.198
Age (years)	–0.01	–0.03–0.01	0.363	–0.03	–0.08–0.02	0.275
Gender (male)	–0.12	–0.87–0.63	0.745	–0.25	–1.90–1.39	0.760
Race (White)	–0.01	–0.78–0.76	0.976	–0.33	–2.01–1.36	0.704
Institution (others)	–0.93	–2.12–0.26	0.123	–1.63	–4.24–0.97	0.217
Location (ICU location vs ED)	0.51	–0.62–1.63	0.376	1.41	–1.06–3.88	0.262
Location (non-ICU location)	1.52	0.57–2.47	**0.002**	4.06	1.97–6.14	**<0.001**
Presence of any comorbidity	–0.21	–1.51–1.08	0.745	–0.02	–2.87–2.82	0.987
Fever	–0.43	–1.23–0.38	0.296	–0.95	–2.70–0.81	0.290
Headache	–0.86	–1.84–0.13	0.089	–1.09	–3.26–1.08	0.323
Altered mental status	0.13	–0.87–1.12	0.802	1.10	–1.08–3.28	0.321
Neck stiffness	0.03	–1.20–1.26	0.963	0.53	–2.16–3.23	0.697
Seizure	–0.16	–1.28–0.95	0.771	–0.31	–2.76–2.13	0.800
Focal neurologic deficits	0.48	–0.54–1.51	0.353	1.08	–1.17–3.33	0.345
CSF WBC over 5	1.34	0.55–2.14	**0.001**	3.31	1.56–5.05	**<0.001**
CSF glucose	–0.00	–0.02–0.01	0.637	0.00	–0.03–0.03	0.990
CSF protein	–0.00	–0.00–0.00	0.995	–0.00	–0.01–0.01	0.826
Positive CSF Gram stain	2.94	0.73–5.15	**0.010**	6.76	1.91–11.62	**0.007**
Positive CSF culture	–1.26	–3.22–0.69	0.204	–3.13	–7.43–1.16	0.152
Observations	193			193		
*R* ^2^/*R* ^2^ adjusted	0.253/0.171			0.277/0.198		

Note. CI, confidence interval; FA/ME, meningitis/encephalitis; CSF, cerebrospinal fluid; WBC, white blood cells; ED, emergency department; ICU, intensive care unit.

## Discussion

Our findings showed that the implementation of FA/ME panel orders did not significantly affect antibiotic prescribing patterns in patients with suspected community-acquired meningitis. Despite the rapid results of the FA/ME panel compared to CSF culture, there was no statistical difference between the duration of empiric antibiotic therapy or DOT when controlling for confounders.^5^

Previous literature showed mixed findings on the impact of the FA/ME panel on antimicrobial usage.^6,7^ Most of the previous literature includes pediatric populations and non-antibiotic antimicrobials such as acyclovir, which may not reflect the utility of the FA/ME panel on antibiotic usage in adult populations.^6,7^ Meningitis presents with different symptoms, causative pathogens, and treatment algorithms in pediatric and adult populations, as well as different severity and treatment with bacterial and viral pathogens, limiting comparison between our study and previous research.^8^ In the systematic review by Goodlet et al. and meta-analysis by Hueth et al., a key limitation was that no studies with adult populations separated the usage of antibiotics and acyclovir, which leads to a strong confounder in how physicians approach viral compared to bacterial meningitis.^6,7^

We found two other studies that tracked specifically antibiotic usage differentiated from total antimicrobial usage in adult populations. Of these studies, one showed a decrease in antibiotic usage, although another showed no change.^9,10^ A key difference between the study by Choi et al., which showed a reduction in antibiotic usage, is that clinicians received education regarding the interpretation of the FA/ME panel, which was not present in our study or the study by Kitagawa et al.^9,10^ This education included institutional emails, conferences, and direct communication of positive results from the Clinical Microbiology Laboratory to the clinician by phone.^9^

Our study adds to the literature, given its focus on empiric antibiotic prescriptions in adults. Our study is also unique in that there was no pre-post design as in most previous studies, possibly limiting the non-contemporaneous control bias in quasi-experimental data.^6,7,9,10^ The lack of impact of the panel at our hospital highlights the need for education and prospective antibiotic stewardship efforts when implementing new diagnostic tests.

Limitations of our study include possible selection bias due to the optional nature of the FA/ME panel orders. To address this bias, we controlled for patient baseline characteristics, presenting symptoms, comorbidities, and location that could affect the decision to order the FA/ME panel. We did not manually review all clinician notes, which could have led to confounding bias from patients receiving antibiotics for non-central nervous system (CNS) infections; however, we aimed to limit this bias by excluding all patients with any other positive non-CNS culture results. Finally, the intervention only included one academic health system, limiting generalizability.

## Conclusion

Implementing the FA/ME panel to evaluate adult patients with suspected community-acquired meningitis did not significantly affect antibiotic prescriptions. Further work should include concurrent active antibiotic stewardship interventions as well as training clinicians on interpreting FA/ME panel results while providing prospective audits and feedback.

## Manuscript preparation

Statistical and other analyses were done by the author MA.
